# Impact of Low Birth Weight on the Prevalence and Economic Burden of Common Childhood Illnesses Among Under-Five Children in India: Findings From Nationally Representative Surveys

**DOI:** 10.7759/cureus.41507

**Published:** 2023-07-07

**Authors:** Sreeja Manghat, Sitanshu Kar, Adhisivam Bethou, Sonali Sarkar

**Affiliations:** 1 Preventive and Social Medicine, Jawaharlal Institute of Postgraduate Medical Education and Research, Puducherry, IND; 2 Neonatology, Jawaharlal Institute of Postgraduate Medical Education and Research, Pondicherry, IND

**Keywords:** nsso, nfhs-5, out-of-pocket expenditure (oope), childhood illness, under-five children, low birth-weight

## Abstract

Background

Common childhood illnesses such as diarrhea, fever, and acute respiratory infection impose substantial health burdens among under-five children, and Low Birth Weight (LBW) has been associated with an increased prevalence of these illnesses. However, the impact of LBW on healthcare utilization and the economic burden of these illnesses remains understudied.

Aim

To assess the impact of LBW on the prevalence, healthcare utilization, and Out of Pocket Expenditure (OOPE) for outpatient (OP) treatment of selected Common Childhood Illnesses (CCHI) among under-five children in India.

Methodology

This study utilized data from two nationally representative surveys conducted in India; National Family Health Survey (NFHS-5) (2019-2021) and the National Sample Survey Organization (NSSO) 75th Round Schedule Social Consumption: Health (2017-2018). Data from the NFHS-5 was analyzed to assess the impact of LBW on the prevalence of selected CCHI and healthcare utilization. Comparison of OOPE for OP treatment of selected CCHI between LBW and Normal Birth Weight (NBW) children done using the median OOPE for OP visits of CCHI estimated from the NSSO data.

Results

The two-week prevalence of selected CCHI among LBW and NBW children was found to be 20.0% (95% CI 19.6 -20.4) and 18.0% (95% CI 17.8 -18.2), respectively. There was no significant difference between LBW and NBW children on healthcare utilization for the treatment of CCHI; both groups had a similar proportion (around 70%) of formal medical treatment utilization for CCHI. The median OOPE spending for OP visits per episode of CCHI was comparable between LBW and NBW children. However, families of LBW children had higher annual OOPE spending for OP visits related to CCHI, with projected estimates of INR 1,446 ($19.56) for LBW children and INR 1,271 ($17.2) for NBW children.

Conclusion

LBW was associated with a higher prevalence of CCHI. Even though healthcare utilization was similar among LBW and NBW children, a higher prevalence of CCHI among LBW children led to higher OOPE. LBW children have approximately 13% higher annual OOPE spending for the OP visits related to selected CCHI compared to NBW children.

## Introduction

Low birth weight (LBW) is a crucial public health indicator that can provide insights into various factors related to child health, nutrition, healthcare delivery, and poverty [1]. It is an important measure for assessing the survival and overall health of newborns [2]. Since birth weight is strongly associated with the health and nutritional status of the mother, it closely reflects the health status of the communities into which they are born [3].

The World Health Organization (WHO) defines LBW as a birth weight of less than 2,500 grams regardless of gestational week at delivery [4]. The estimated global prevalence of LBW in 2015 was 14.6%, with the highest prevalence (26.4%) reported from Southern Asia [5]. A more recent report, the National Family Health Survey (NFHS-5) (2019-2021), indicates that the prevalence of LBW in India is 18.2% [6].

LBW can challenge the health of children in a number of ways; children born with LBW had a higher risk of mortality and morbidity, malnutrition in childhood, certain child malignancies, developmental delays, etc. [7-11]. LBW is also associated with long-term physical ill health in adulthood, which includes cardiovascular disease, renal diseases, psychiatric disorders, cardiometabolic diseases, etc. [12-13].

Common childhood illnesses such as fever, acute respiratory infections (ARI), and diarrhoea are the major contributors to the under-five mortality rate [14]. The 2020 global disease burden study revealed that lower respiratory tract infections and diarrhoea are the second and third leading causes of Disability-Adjusted Life Years (DALYs) among children aged 0 to 9 years in 2019 [15]. A cohort study done in India from 2014 to 2016 found that respiratory tract infections, gastrointestinal illness, and fever are the highest reported illness among children during the first two years and accounted for approximately 74% of all reported morbidity episodes [16].

Several studies conducted globally have found that LBW is associated with a higher incidence of childhood infections [11,17-19]. National Health Account Estimates in India for 2019-20 reported that 52% of healthcare expenditures are met through out-of-pocket spending [20]. A study on data analysis of the National Sample Survey Organization (NSSO) 75th schedule reported that the OOPE for outpatient (OP) care and hospitalization in households reporting childhood infections were 4.8% and 6.7% of their total consumption expenditure in India [21]. Childhood infections lead to poor health outcomes and impose a significant financial burden on households and society [21]. Since LBW is strongly associated with a higher incidence of childhood illnesses and long-term health issues, which can have significant financial implications for households and healthcare systems, particularly in low- and middle-income countries.

Although many studies worldwide have shown a strong association between LBW and childhood morbidity [11,17-18], there has been limited research exploring the financial implications associated with it. Many of these studies were conducted as small-scale cohort studies. In this study, we aim to assess the impact of LBW on the prevalence of selected common childhood illnesses, healthcare utilization, and OOPE spending of this illness by analyzing data from two nationally representative surveys such as NFHS-5 and NSSO 75th Schedule for Social Consumption Health. Our research has the potential to provide valuable insights into the influence of LBW on the prevalence of CCHI, healthcare utilization, and the additional financial burden placed on households due to the management of these illnesses for LBW children.

## Materials and methods

Sources of data

This study utilized data from two nationally representative surveys conducted in India.

NFHS-5 Survey (2019-2021)

The NFHS-5 survey encompassed 636,699 households, 724,115 women, and 101,839 men from all states and Union Territories (UT) of India. The survey utilized a two-stage, stratified cluster sampling design and covered topics on fertility, mortality, family planning, maternal and child health, reproductive health, nutrition, anemia, and the utilization and quality of healthcare services, etc. The detailed NFHS -5 survey methodology was provided elsewhere [6].

NSSO 75th Round for Schedule Social Consumption: Health (2017-18)

The NSSO 75th schedule survey covered 1,13,823 households and 5,55,115 persons from all the states and U.T.s from India. A stratified multi-stage design was adopted for this survey. The survey collected data on health indicators like disease prevalence, healthcare utilization, expenses, and socio-economic characteristics such as income, education, and occupation. The detailed NSSO 75th schedule methodology was published elsewhere [22].

Study variables and operational definitions

We have included children who have completed the age range of 0 to 4 years from both surveys as under-five children. Infants with a birth weight below 2,500g are classified as LBW. To ensure data accuracy, infants with missing or implausible birth weight measurements (birth weight less than 500g or more than 6,000g) were excluded from the study. As the NSSO survey did not capture the birth weight of children, birth weight classification was only possible in NFHS-5 data.

Both surveys collected data on the two-week prevalence of common childhood illnesses and healthcare utilization. The NFHS-5 survey captured data on the occurrence of three major common childhood illnesses such as fever, diarrhoea, and symptoms of respiratory illness such as cough, shortness of breath, and chest problems. In NFHS-5, participants were asked to recall whether the child had experienced any of these illnesses during the last two weeks and indicate their responses as “Yes" (present), "No" (not present), or "Don't Know" (unknown). Whereas the NSSO survey recorded the occurrence of various ailments during the last 15 days recall period by classifying them into 60 different health conditions without specific responses for each morbidity. Both NFHS-5 and NSSO followed a similar operational definition for diarrhoea which includes the passage of three or more liquid stools per day. The operational definitions for ARI were slightly different in both surveys, NFHS-5 operational definitions of ARI included the presence of short, rapid breathing or difficult breathing which were chest-related whereas NSSO definitions of ARI included the presence of running nose, cough, and sore throat, with or without fever all of the short duration, though it could be recurrent or cough as the main symptom, with or without fever, with or without sputum and blood in it, with or without marked breathlessness. To harmonize the operational definitions of fever between the two surveys, we have categorized fever without any accompanying symptoms of respiratory illness in the NFHS-5 survey as "Fever". For this study, the presence of any of these three morbidities (diarrhoea/ARI/fever) is classified as Common Childhood Illness (CCHI).

Both the NFHS-5 and NSSO surveys provided information on healthcare utilization for CCHI. The healthcare utilization patterns for CCHI were evaluated based on the type of treatment received, categorized as no treatment, informal treatment, treatment in public facilities, and treatment in private facilities. In the NSSO survey dataset, informal treatment is directly recorded and defined as treatment provided by untrained health service providers working outside regulatory frameworks. On the other hand, in the NFHS-5 survey, responses for informal treatment were derived from a set of variables, which included treatment from pharmacies, paramedical personnel, shops, friends or relatives, home remedies, and other non-formal treatments. NSSO survey provided direct responses for treatment taken in public health facilities which included all public institutions such as sub-centers, primary health centers (PHCs), community health centers (CHCs), etc. In NFHS 5 treatment from the public facility was derived from a set of variables such as government hospitals, government AYUSH, PHCs, CHCs, sub-centers, government mobile clinics, public camps, and anganawadi. The NSSO survey provided a direct response for treatment taken in private facilities which included private doctors or clinics and private hospitals. In the NFHS-5 survey treatment from the private facility was derived from a set of variables which included treatment from a private hospital/clinic, private doctor, private AYUSH, and other private institutions. In this study, since the number of individuals receiving treatment from NGOs or charitable institutes was low in both surveys, these facilities have been included in the private facility category for the purpose of analysis. An outcome variable, a formal medical treatment created in both surveys, refers to treatment sought in either private or public facilities.

NSSO survey provided details of OOPE for CCHI whereas NFHS-5 did not collect information on OOPE for CCHI. We defined Direct OOPE as spending on doctor/surgeon fees, medicines, diagnostic services, and other medical expenses and Indirect OOPE as expenditure on transportation fees, expenditure for food, etc.

Covariates

The NFHS-5 data set was used to assess the other factors related to the prevalence of CCHI and healthcare utilization. These included the sex of the child (male/female), age of the children was expressed in completed months and classified into four groups (less than six months, 6 to 12 months, 13 to 36 months, and 37 to 59 months). The birth order of the child was categorized as one, two to three, or more than three. Place of delivery was grouped into three categories: home delivery, delivery in a government facility, and delivery in a private facility. The type of delivery was classified as either caesarean or vaginal. The place of residence was categorized as urban or rural. The social group included categories such as schedule caste, schedule tribe, other backward castes, and others. Religion was classified as Hindu, Muslim, Christian, and others. The age of the mother was expressed in completed years and grouped into four categories: 15 to 20 years, 21 to 29 years, 30 to 39 years, and 40 years and above. The education of the mother was categorized as no education, primary education (grades 1 to 5), secondary education (grades 6 to 12), and higher education (grades 12 and above). The wealth index, a composite measure calculated from household assets and durable possessions, was divided into quintiles ranging from 1 (poorest) to 5 (richest) [6]. Indian States and Union Territories (UT) grouped based on the Social Progress Index (SPI) which evaluated the progress of states and districts based on 12 components related to basic human needs, foundations of well-being, and opportunities. Based on the SPI index States/UTs were classified into high, middle, and low social progress categories [23]. Covariates were selected based on the factors associated with morbidity and related expenditures known from the literature and available in the datasets.

Statistical analyses

All the statistical analyses were done using STATA version 14.0 (Stata Corp LP, College Station, TX, USA). Sample weights provided with the NFHS and NSSO data set were used for descriptive analysis. All the percentages reported in the study are weighted percentages. To account for the complex survey design, the 'svy" suite command was used in the NFHS -5 analysis for the Pearson Chi-square test to assess the difference in the prevalence of CCHI and healthcare utilization for CCHI with selected LBW covariates. Unweighted multiple binary logistic regression was used to assess LBW's association with the prevalence of CCHI and formal medical treatment. The Median OOPE estimates, calculated from NSSO data, were adjusted for inflation to 2021 price using the Consumer Price Index (CPI) and these estimates were further converted to United States dollars (US $) using the average annual exchange rate of 2021 (1 US $=₹73.92).

$Adjusted\ median\ \ OOPE\ in\ 2021=Median\ OOPE\ in\ 2018\ (NSSO\ estimates)\ \times\ \frac{CPI\ for\ 2021}{CPI\ for\ 2018}$

The OOPE for an average episode of illness and annual average OOPE spending for CCHI among LBW and NBW children was calculated by the following formulas. To project the two-week OOPE estimates to annual OOPE estimates we have applied a multiplication factor of 26.1 (52 weeks/2). The annual projected OOPE was calculated by assuming the same patterns of prevalence of CCHI and health expenditure throughout the year.

$Projected\ OOPE\ for\ an\ episode\ of\ CCHI\ =\frac{(No.of\ children\ Utilzed\ public\ facility\times M e d i a n\ OOPE\ in\ public)+(No.of\ children\ Utilzed\ Pvt\ facility\times\ Median\ OOPE\ in\ private)}{Number\ of\ children\ with\ CCHI}$

$Projected\ annual\ estimates\ of\ OOPE\ for\ CCHI=\frac{Total\ projected\ estimates\ of\ OOPE\ for\ CCHI\times26.1}{Total\ Number\ of\ children}$

To apply this formula, we used median adjusted OOPE estimates from NSSO data and health care utilization percentage for CCHI, the number of children with CCHI, and the total number of children from the NFHS-5 data.

## Results

**Figure 1:**
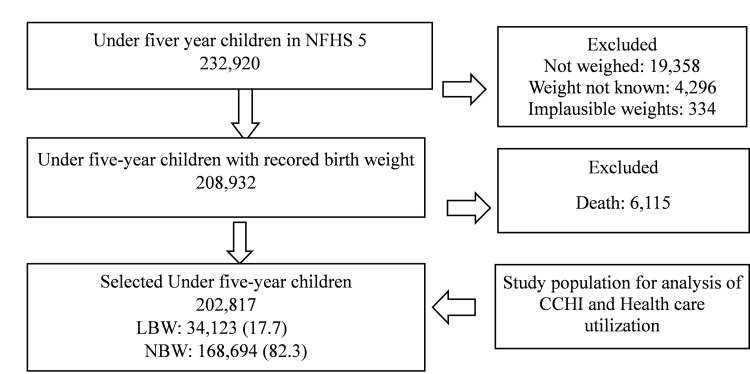
Selection of children for the NFHS-5 survey analysis NFHS-5: National Family Health Survey (2019-2021)

Figure 1 shows the selection of under-five children from NFHS-5 survey data for analyzing the impact of LBW on the prevalence of CCHI and treatment-seeking patterns. About 7.5% of children were not weighed at birth, and for nearly 2% of children, birth weight was not known to mothers. The percentage of under-five children with Extremely Low Birth Weight (ELBW) and Very Low Birth Weight (VLBW) was 0.06% (n =106) and 0.95% (n=1,683) respectively. A total of 202,817 children were included in the analysis. The Mean (SD) birth weight of selected LBW and NBW children were 2007g (312) and 2980g (428) respectively.

Association of LBW on the prevalence of CCHI

Table 1 describes the prevalence of CCHI with LBW and selected covariates. The association between LBW with the prevalence of CCHI is presented in Table 2. We have observed that LBW children had a higher prevalence of all CCHI compared with NBW children.

**Table 1:** Prevalence of CCHI among children with LBW and other characteristics *All n are unweighted CCHI: Common Childhood Illnesses; LBW: Low Birth Weight

Variables		Total (N)	CCHI		P value
			Frequency (n)	Percentage	
Birth weight category	LBW (<2500 gms)	34,123	6,623	20.0	<0.001
	NBW(>=2500gms)	168,694	28,969	18.0	
Sex	Male	105,083	19,049	19.0	<0.001
	Female	97,734	16,543	17.6	
Age of the children (in months)	< 6 months	21,184	3,375	17.1	<0.001
	6 to 12 months	23,610	5,693	25.9	
	13 to 36 months	80,039	1,547	20.1	
	37 to 59 months	77,984	11,077	14.6	
Birth order of the child	1	80,752	14,002	18.1	0.006
	2 to 3	98,924	17,228	18.3	
	> 3	23,141	4,362	19.4	
Place of delivery	Home delivery	15,060	2,791	18.8	0.562
	Government facility	140,916	24,388	18.4	
	Private facility	46,433	8,310	18.2	
Mode of delivery	Vaginal	160,327	27,417	18.1	0.003
	Caesarean	42,490	8,175	19.1	
Place of residence	Urban	43,308	7,201	16.6	<0.001
	Rural	159,509	28,391	19.0	
Social group	Schedule Caste	41,205	7,457	18.8	0.002
	Schedule Tribe	38,908	6,601	17.5	
	Other Backward Cast	78,688	13528	17.8	
	Others	33,130	5834	19.2	
Religion	Hindu	151,447	26,042	18.1	<0.001
	Muslim	28,563	5,207	19.7	
	Christian	14,461	2,904	21.1	
	Others	8,346	1,439	16.2	
Age of the mother (years)	15 to 20	11,382	2,700	25.0	<0.001
	21 to 29	133,211	23,570	18.7	
	30 to 39	54,131	8,643	16.0	
	40 and above	4,093	679	15.2	
Education of the mother	No education	39,196	6,407	17.6	<0.001
	Primary	24,920	4,611	19.0	
	Secondary	108,372	19,860	19.4	
	Higher	30,329	4,714	15.5	
Wealth Index	Poorest	49,196	9,218	20.4	<0.001
	Poor	46,870	8,656	19.6	
	Middle	40,964	7,224	18.9	
	Richer	3,6404	6,176	17.2	
	Richest	29,383	4,318	14.7	
State group based on SPI	High social progress	29,269	4,051	14.3	<0.001
	Middle social progress	88,415	16,191	18.5	
	Low social progress	85,133	15,350	19.2	

**Table 2:** Association between LBW with the prevalence of selected CCHI #Variable adjusted are child age, sex, birth order, social group, religion, place of residence, mother age, education of mother, state category based on SPI, wealth index and type of delivery * All n are unweighted CCHI: Common Childhood Illnesses; LBW: Low Birth Weight; NBW: Normal Birth Weight

Ailments	LBW n (%)	NBW n (%)	Unadjusted Odds ratio (95% CI)	Adjusted odds ratio (95% CI)
Diarrhoea	2,703 (8.0)	11,266 (7.1)	1.20 (1.15-1.25)	1.16 (1.11-1.21)
ARI	1,088 (3.2)	4,457 (2.7)	1.21 (1.13-1.29)	1.18 (1.10-1.26)
Fever	3,932 (12.3)	17,772 (11.1)	1.11 (1.07-1.15)	1.01 (1.05-1.14)
Common Childhood Illness (CCHI)	6,623 (20.1)	28,969 (18.0)	1.16 (1.12-1.19)	1.14 (1.10-1.17)

Relationship between LBW and healthcare utilization pattern for CCHI

Table 3 compares the healthcare utilization pattern of LBW and NBW children for CCHI. In both groups medical treatment was sought for nearly 70% of CCHI. The highest medical treatment was sought for fever (74%) and the least for ARI (51.6%). We observed that many socio-economic background characteristics were highly associated with the utilization of formal medical treatment (Table 4).

**Table 3:** Comparison of healthcare utilization of LBW and NBW children for CCHI *All n are unweighted * Multiple responses possible LBW: Low Birth Weight; NBW: Normal Birth Weight

Type of Healthcare utilization	LBW N (%)	NBW N (%)	Total N (%)	P value
Diarrhoea				
No treatment	650 (23.0)	2,653 (22.5)	3,303 (22.6)	0.6586
Informal treatment	217 (7.65)	967 (8.63)	1,184 (8.44)	0.3978
Public facility	809 (24.6)	3,456 (25.8)	4,265 (25.6)	0.3059
Private facility	1,073 (46.3)	4,491 (45.9)	5,564 (46.0)	0.7583
Formal Medical treatment	1,868 (70.5)	7,830 (70.6)	9,698 (70.6)	0.9073
ARI				
No treatment	89 (12.5)	306 (11.3)	395 (11.5)	0.4179
Informal treatment	65 (6.5)	183 (4.4)	248 (4.8)	0.0125
Public facility	223 (15.6)	986 (17.7)	1,209 (17.3)	0.1165
Private facility	318 (37.7)	1,296 (34.6)	1,614 (35.3)	0.2099
Formal Medical treatment	537 (53.1)	2,238 (51.2)	2,775 (51.6)	0.4081
Fever				
No treatment	840 (20.0)	3,839 (19.8)	4,679 (19.8)	0.7781
Informal treatment	241 (5.8)	1,210 (7.0)	1,451 (6.7)	0.0319
Public facility	1,101 (24.3)	5,027 (22.7)	6,128 (23.0)	0.1039
Private facility	1,745 (50.0)	7,678 (50.7)	9,423 (50.6)	0.5236
Formal Medical treatment	2,804 (73.2)	12,524 (72.3)	15,328 (72.4)	0.4218
CCHI				
No treatment	1,479 (22.3)	6,421 (21.9)	7,900 (22.0)	0.6286
Informal treatment	473 (6.9)	2,181 (7.7)	2,654 (7.5)	0.0678
Public facility	1,919 (24.6)	8,461 (24.1)	10,380 (24.2)	0.4520
Private facility	2,731 (47.6)	11,832 (47.4)	14,563(47.7)	0.8974
Formal Medical treatment	4,553 (70.8)	19,821 (70.1)	24,374 (70.2)	0.3878

**Table 4:** Prevalence of Formal Medical treatment utilization for CCHI by Background Characteristics *All n are unweighted

Variables		Total number of children with CCHI (N)	Number of children availed medical treatment		P value
			Frequency (n)	Percentage	
Sex	Male	19,049	13,199	70.8	0.048
	Female	16,543	11,175	69.6	
Age of the children (in months)	< 6 months	3,375	2,212	65.4	<0.001
	6 to 12 months	5,693	4,071	71.5	
	13 to 36 months	15,447	10,755	69.6	
	37 to 59 months	11,077	7,336	66.2	
Birth order of the child	1	14,002	9,589	70.4	0.026
	2 to 3	17,228	11,871	70.6	
	> 3	4,362	2,914	67.8	
Place of delivery	Home delivery	2,791	1,743	64.8	<0.001
	Government facility	24,388	16,461	69.0	
	Private facility	8,310	6,100	74.4	
Delivery type	Vaginal	27,417	18,483	69.2	<0.001
	Caesarean	8175	5,891	73.2	
Place of residence	Urban	7,201	5,137	73.9	<0.001
	Rural	28,391	19,237	69.1	
Social group	Schedule Cast	7,457	5,132	69.4	<0.001
	Schedule Tribe	6,601	4,266	68.3	
	Other Backward Cast	13,528	9,515	70.4	
	Others	5,834	4,102	73.0	
Religion	Hindu	26,042	18,013	69.9	0.054
	Muslim	5,207	3,615	72.0	
	Christian	2,904	1,821	67.3	
	Others	1,439	925	69.8	
Age of the mother	15 to 20	2,700	1,820	68.0	0.020
	21 to 29	23,570	16,245	70.6	
	30 to 39	8,643	5,872	70.2	
	40 and above	679	437	63.8	
Education of the mother	No education	6,407	4,217	66.5	<0.001
	Primary	4,611	3,106	69.0	
	Secondary	19,860	13,689	71.0	
	Higher	4,714	3,362	73.0	
Wealth Index	Poorest	9,218	5,844	64.8	<0.001
	Poor	8,656	5,836	68.4	
	Middle	7,224	5,064	71.8	
	Richer	6,176	4,500	75.0	
	Richest	4,318	3,130	74.3	
State group based on SPI	High social progress	4,051	2,761	74.0	<0.001
	Middle social progress	16.191	11,675	74.3	
	Low social progress	15,350	9,938	65.5	

Treatment seeking pattern and OOPE for OP treatment of selected CCHI from NSSO survey analysis

NSSO 75th schedule survey contains data from 64,732 under-five children. The proportion of under-five children who reported any episodes of illness (both acute and chronic) and acute episodes of illness during the last 15 days were 8.5% (n:4435) and 8.2% (n:4259) respectively. A total of 4459 episodes of acute illnesses were reported from 4259 under-five children. Among these 3898 episodes of illness (87.4%) were selected as CCHI. We have noted that formal medical treatment was sought for nearly 79% of selected CCHI (Table 5). Estimates of OOPE for OP treatment of CCHI are presented in Table 6. The mean OOPE for OP visits of CCHI is 540₹, and the mean OOPE for OP treatment of CCHI in Public and Private facilities was 327 ₹ and 668 ₹, respectively.

**Table 5:** Treatment seeking pattern for selected CCHI: results from NSSO survey analysis *All n are unweighted ARI: Acute Respiratory Infections; CCHI: Common Childhood Illnesses; NSSO: National Sample Survey Organization

Illness	Total number of episodes N	Type of Healthcare utilization for CCHI n (%)					
		No treatment/not taken treatment on medical advice	Informal treatment	Treatment in a public facility	Treatment in a private facility	Formal Medical treatment	Hospitalisation
Diarrhoea	225	26 (18.1)	7 (1.8)	57 (23.5)	135 (56.6)	192 (80.1)	11 (2.15)
ARI	1,016	153 (19.4)	29 (7.8)	223 (19.2)	611 (53.6)	834 (72.8)	8 (0.23)
Fever	2,657	327 (14.3)	96(5.3)	545 (20.9)	1,689 (59.6)	2,234 (80.4)	35 (0.31)
CCHI	3,898	506 (15.6)	132 (5.7)	825 (20.6)	2,435 (58.1)	3,260 (78.7)	54 (0.39)

**Table 6:** OOPE for OP treatment of CCHI *Adjusted for inflation to 2021 prices OOPE: Out of Pocket Expenditure; CCHI: Common Childhood Illnesses

Illness	Facility-wise OOPE for OP treatment per episode of illness in ₹ Median (IQR)				Adjusted OOPE per episode of illness in $*	Total OOPE of OP treatment for per episode of illness in ₹ Median (IQR)			Total Adjusted OOPE in 2021 $*
	Facility type	Direct	Indirect	Total		Direct	Indirect	Total	
Diarrhoea	Public	130 (0-300)	50 (0-150)	200 (110-322)	3.14	400 (150-720)	50 (0-150)	470 (200-810)	7.38
	Private	520 (235-850)	50 (0-150)	610 (300-960)	9.57				
ARI	Public	100 (0-260)	30 (0-80)	150 (40-320)	2.35	290 (130-500)	40 (0-80)	327.5 (165-590)	5.14
	Private	350 (200-570)	40 (0-90)	400 (225-670)	6.28				
Fever	Public	100 (0-300)	50 (20-120)	200 (70-410)	3.14	350 (170-600)	50 (0-100)	410 (200-700)	6.43
	Private	400 (250-650)	50 (0-100)	480 (285-75)	7.53				
CCHI	Public	100 (0-300)	50 (5-125)	180 (65-390)	2.82	330 (150-580)	41 (0-100)	400 (200-674)	6.28
	Private	400 (230-650)	40 (0-100)	470 (260-750)	7.38				

Estimation of OOPE for OP treatment of CCHI among LBW and NBW children: projection of NSSO estimates on OOPE to NFHS-5 results of prevalence and healthcare utilization for CCHI among LBW and NBW children

For comparing OOPE for OP treatment of CCHI among LBW and NBW children, we have applied NSSO, OOPE estimates of CCHI to NFHS-5 results of prevalence and health care utilization pattern of CCHI (Table 7). We did not find much difference in per episode OOPE for OP treatment of CCHI between LBW and NBW children as they have similar health care utilization. Since the episodes of CCHI were more among LBW children, the total OOPE incurred by LBW children was much higher than NBW children. We have calculated the average annual OOPE for OP treatment of CCHI and found that LBW children have an average annual OOPE for outpatient visits of $19.56 (INR 1,446), while NBW children have an average annual OOPE spend of $17.2 (INR 1,271). This suggests that the average annual OOPE spending for OP treatment of CCHI among LBW children is approximately 13% higher compared to NBW children.

**Table 7:** Projected estimates of OOPE for CCHI among LBW and NBW children OOPE: Out of Pocket Expenditure; CCHI: Common Childhood Illnesses; LBW: Low Birth Weight; NBW: Normal Birth Weight

CCHI	Prevalence of CCHI*		Facility of health care utilization*			Total Median OOPE $	Projected OOPE facility-wise $		Total projected OOPE$		Total projected OOPE per episode of CCHI $		Average annual OOPE spending for CCHI $	
	LBW n (%)	NBW n (%)	Facility type	LBW n (%)	NBW n (%)		LBW	NBW	LBW	NBW	LBW	NBW	LBW	NBW
Diarrhoea	2,703 (8.0)	11,266 (7.1)	Public	809 (24.6)	3,456 (25.8)	3.14	2,540.26	10,851.84	12,808.87	53,830.71	4.74	4.78	9.80	8.33
			Private	1,073 (46.3)	4,491 (45.9)	9.57	10,268.61	42,978.87						
ARI	1,088 (3.2)	4,457 (2.7)	Public	223 (15.6)	986 (17.7)	2.35	524.05	2,317.1	2,521.09	10,455.98	2.32	2.35	1.93	1.62
			Private	318 (37.7)	1,296 (34.6)	6.28	1,997.04	8,138.88						
Fever	3,932 (12.3)	17,772 (11.1)	Public	1,101 (24.3)	5,027 (22.7)	3.14	3,457.14	15,784.78	16,596.99	73,600.12	4.22	4.14	12.69	11.39
			Private	1,745 (50.0)	7,678 (50.7)	7.53	13,139.85	57,815.34						
CCHI	6,623 (20.1)	28,969 (18.0)	Public	1,919 (24.6)	8,461 (24.1)	2.82	5,411.58	23,860.02	25,566.36	111,180.2	3.86	3.84	19.56	17.20
			Private	2,731 (47.6)	11,832 (47.4)	7.38	20,154.78	87,320.16						

## Discussion

In this study, we have assessed the impact of LBW on prevalence, healthcare utilization, and OOPE for the OP treatment of CCHI in India. This study utilized data from two large-scale national representative surveys; the NSSO 75th Schedule of social consumption health and the NFHS-5 survey.

Based on our analysis of the National Family Health Survey-5 data, we have observed a higher prevalence of all CCHI in children with LBW compared to those with NBW. Despite the relatively short recall period (2 weeks), the large sample size of the NFHS-5 survey provides robust estimates of the association between LBW and CCHI. Our findings align with several prior studies that have also reported an increased prevalence of CCHI in LBW children [11,17-18]. These results indicate that LBW constitutes a significant risk factor for the development of CCHI in children, emphasizing the necessity for targeted interventions to enhance the health outcomes of this vulnerable population.

Our analysis of the NFHS-5 data revealed that the proportion of formal medical treatment utilization for CCHI was 70%. However, this estimate is lower than the analysis from the NSSO survey, which reported a higher utilization of formal medical treatment for CCHI (79%). This difference in estimates could be attributed to various factors, such as differences in the survey methodologies, sample sizes, and population characteristics. However, despite these differences, the results of these surveys can be considered comparable as both surveys covered a large representative sample of households in India and are similar to results from other smaller studies in various parts of India. A cohort study conducted in the rural area of Pune, India, in 2015-16 reported that formal treatment was sought for 70% of episodes of illness among under-five children [24]. Similarly, a cross-sectional study of tribal children from south India also reported that 70% of under-five children sought formal medical treatment [25]. A higher prevalence of seeking formal treatment (84%) was observed from a cross-sectional study done in the urban slums of Orissa, India in 2016-17 [26]. These differences in the prevalence of formal medical treatment can be attributed to differences in settings and various other cultural and health system factors.

Results of this study show no difference in the healthcare utilization between LBW and NBW children; although a good sign, does not reflect the anxiety of families having children with LBW, which would lead to higher healthcare utilization. On the contrary, a cohort study from Ghana in 2013 reported a higher proportion of absence of care seeking among LBW children compared with NBW children [27]. This is expected given the factors leading to LBW also contribute to poor health-seeking behaviors.

We estimated the median OOPE for OP treatment of selected CCHI in India using the NSSO 75th schedule dataset. While there is a published study that analyzed the same dataset to assess the average OOPE per episode of outpatient visit and hospitalization in children aged less than five years for selected infectious diseases [21], they reported the average OOPE for outpatient visits and hospitalization for common childhood illnesses. We have conducted a different analysis using the same dataset to estimate the median OOPE for outpatient visits of three selected common childhood illnesses. In our study, we have found that the median OOPE for OP treatment of diarrhoea, fever, and ARI was 470₹, 328₹, and 410₹ respectively. We also estimated the median OOPE for OP treatment of all three selected CCHI together as 400₹. Similar to our finding a study from slums of eastern India in 2019 reported the median OOPE for outpatient visits of childhood illness as 326₹ [28] and another study from urban Puducherry, on the southeast coast of India in 2015, reported the median OOPE for childhood illness as 375₹ [29].

In our study, we aimed to compare the OOPE for OP treatment between LBW and NBW children. To achieve this, we applied the adjusted median OOPE of CCHI derived from our analysis of the NSSO dataset to the NFHS results on the prevalence and healthcare utilization patterns of CCHI. Despite high prevalence rates of CCHI among LBW children, we did not find any significant difference in healthcare utilization patterns between the two groups. Therefore, the average OOPE for outpatient visits per episode of illness was similar for both groups. However, due to the higher prevalence of CCHI among LBW children, the total OOPE incurred for this group was greater than that of NBW children. A cohort study from Mozambique found significantly higher household spending for health care costs of LBW infants compared to NBW infants. A report of the NSSO 75th schedule survey on health found that over half of the households (52 percent) faced catastrophic health expenditure when seeking treatment for their one-to-two-year-old children at private hospitals [30].

The present study contributes to the limited literature on the comparison of the OOPE for CCHI between LBW and NBW children in India by providing comparative estimates for outpatient visits for selected CCHI. The use of two nationally representative surveys enhances the generalizability of our findings to the larger Indian population. We have also taken care of the inflation while applying the OOPE estimates from the 2017-18 survey to the 2019-21 survey by calculating the expenditures for 2019-2 with the use of a multiplication factor based on the Consumer Price Indices for these years. However, there are some limitations to our study. Firstly, as a secondary data analysis, we were able to analyze only three selected CCHI, and the estimates may not be representative of the overall burden of childhood illnesses in India, which is likely to be higher increasing the difference estimated between the two groups. Additionally, the prevalence estimates were based on a shorter duration, which could also be viewed as an advantage in reducing the recall bias. However, the other major shortcomings may be the variations in the operational definitions of illness and healthcare utilization of these two surveys. Furthermore, as we only analyzed outpatient visits, our findings do not provide information on the cost of inpatient visits, which are typically more expensive than outpatient visits. If included, this would only inflate the differences observed in the OOPE between the LBW and NBW children.

## Conclusions

Our study showed that LBW children had a higher prevalence of CCHI compared with NBW children. We have also observed higher OOPE spending for OP treatment related to CCHI among LBW children than NBW children. Although our study focused on a limited duration of two weeks and OP visits, the impact of LBW on CCHI prevalence was still evident. Despite numerous studies examining the relationship between LBW and morbidities, very few have explored the financial burden of LBW. Our findings emphasize the need for further research on the financial implications of LBW at the individual and household levels.
